# An Infrared Stripe Noise Removal Method Based on Multi-Scale Wavelet Transform and Multinomial Sparse Representation

**DOI:** 10.1155/2022/4044071

**Published:** 2022-05-30

**Authors:** Mingxuan Li, Shenkai Nong, Ting Nie, Chengshan Han, Liang Huang

**Affiliations:** ^1^Changchun Institute of Optics, Fine Mechanics, and Physics, Chinese Academy of Sciences, Changchun 130033, China; ^2^University of Chinese Academy of Sciences, Beijing 100049, China

## Abstract

The non-uniformity present in the infrared detector and readout circuit leads to significant stripe noises in the infrared images. The effect of these stripe noises on infrared images brings trouble to the subsequent research. The currently available algorithms for removing infrared streak noises cannot effectively protect the non-stripe information while removing the stripe noise. Compared with these algorithms, our algorithm uses a multi-scale wavelet transform to concentrate the streak noise by frequency into vertical components of different scale levels. Then, our algorithm analyzes the unique properties of the streak noise compared to the ideal vertical component. The denoising model of the vertical component at each level is established with its multinomial sparsity, and the streak noise is removed by the alternating direction method of multipliers (ADMM) algorithm for optimal calculation. To prove the usefulness of our algorithm, we carried out a large series of real experiments, comparing it with the most advanced algorithms in terms of both subjective determination and objective indices. The experimental results fully demonstrate the superiority and effectiveness of our algorithm.

## 1. Introduction

Due to the craft as well as the material, non-uniformity exists between the readout circuits of each channel in the infrared detector. These characteristics are reflected in the image as stripe noise, which degrades the image quality and creates resistance to subsequent studies [1–3], such as image recognition, image recovery, and target classification. Therefore, it is significant to investigate the ways to remove the stripe noises as well as retain the detailed information in the infrared images. The main idea of this paper lies in maximizing the extraction of stripe noise from infrared images and maximizing the retention of all information in the images.

Until now, stripe noise removal algorithms can be divided into three main categories after scholars' research: filtering processing method, statistical matching method, and model optimization method.

The earliest application of the filter processing method dates back to 1987 when Quarmby used the a priori knowledge of streak noise in the frequency domain to design filters to remove the streak noise [4]. As the research deepens, wavelet transform gradually plays an increasingly important role in the filter processing method [5–8]. These filtering methods based on wavelet transform take advantage of the wavelet transform's ability to extract local frequencies in an image to make a distinction between high-frequency components and low-frequency components, and then further eliminate stripe noises. Although this type of filtering processing method has the advantages of a simple algorithm and easy application, there are still two shortcomings: On the one hand, this kind of method can extract the stripe noise from the original image only when the stripe noise has strong regularity and does not lose the effective information; on the other hand, this kind of method is easy to mistake the edge information and texture information when the target information is complex, and the original information is lost, and even “artifacts” will be produced.

The statistical matching method is widely adopted in engineering because of its simple algorithm and high operational efficiency. The application of this type of method can be traced back to the processing of MOS-B ocean image data as early as [9]. As an example, the method considers each image element in the detector to have the same average output level for the same radiation input. From there, the corresponding correction factor for each pixel is calculated, including the gain factor and bias factor. These correction coefficients are used to obtain the denoised image. With the development of this type of method, it has been gradually refined by scholars according to the application scenarios. For example, the local constant statistics method combined with neural networks [10]. This method assumes that the mean and variance of each row of pixels using different channel readout circuits are equal, and uses these as the expected values of local statistics to correct for streak noise in the image. The local column equalization method combined with wavelet transform is also one of the methods that emerged during the development process [11]. This method concentrates the streak noise components in the vertical components of each level of the wavelet transform and then removes the streak noise by the column equalization method. Although the above three methods have achieved excellent results in their respective application scenarios, it is difficult to remove the noise satisfactorily in the case where the streak noise presents an uneven distribution in the image.

The model optimization method, as one of the most advanced methods in the current field of infrared image streak noise removal, is based on the establishment of mathematical models and is widely adopted by scholars. The use of this class of methods can be traced back to 2004 when Antonin Chambolle constructed the cost function according to the ideal image characteristics and found the denoised image that minimizes the cost function by the steepest gradient descent method [12]. Later, some scholars built on this model and introduced a better denoising effect by using the L1 parametric description of the grayscale difference between the ideal image and the original image [13]. Some other scholars combined the three major types of methods, used frequency domain filtering to extract the streak noise component at high frequencies, and constructed a mathematical model with the gradient equilibrium of the ideal image to finally achieve the streak noise removal [14]. Currently, the most representative algorithm is the streak noise removal model based on group sparsity. The algorithm uses the L1 parametric representation of group sparsity of streak noise and obtains a better denoising effect [15].

After analyzing the above three major types of infrared image stripe noise removal of algorithms, we can learn that the goal of current algorithms tends to focus on the removal of stripe noise, and little consideration is given to the information loss in non-stripe noise regions. This leads to the current phenomenon that there is still a slight streak noise in the denoised image, and the removal effect is not good, or some information is lost in the denoised image, which reduces the image quality. In the model optimization method, scholars regard the stripe noise as additive noise. And the noise removal problem is converted into the noise extraction problem, in other words, the noise estimation problem [16–18]. To solve this status quo, based on previous research, this paper will aim to remove streak noise while ensuring that the information in the non-streak noise region is not affected, fully exploit the a priori information of the streak noise, and propose practical stripe noise removal algorithms for infrared images.

In this paper, we analyze the proposed algorithm with the example of streak noise in the vertical direction. The stripe noise removal in the horizontal direction is the same as the stripe noise removal in the vertical direction.

Firstly, multi-scale wavelet transform, which can separate spatial information and frequency characteristics into vertical components of different scale levels, is adopted to preserve non-stripe noise information. Moreover, the streak noise is concentrated in the vertical component of each level. Due to its special structuredness, there is a clear distinction between the streak noise and the ideal vertical component at all levels. Then, due to the significant convexity and sparsity of the L1-norm, it is used to construct the optimal model of streak noise in this paper. This method is also known as sparse representation. Finally, this paper presents a new infrared stripe noise removal method based on multi-level wavelet transform and multinomial sparse representation, capable of effectively preserving non-stripe noise information. In addition, the alternating direction method of multipliers (ADMM), a classical method for solving decomposable convex optimization problems, is applied to solve the model in this paper [19, 20]. The proposed method has the following advantages:

1. In this paper, we utilize the multi-scale wavelet transform to separate most of the non-stripe noise information into other components of the different scale levels except the vertical component, which can effectively protect this information from being smoothed by the algorithm.
2. In this paper, we utilize the sparsity and convexity of the L1-norm to construct an algorithmic model with a sparse representation of the gradient sparsity and global sparsity specific to the stripe noise in the vertical component of each level. This is able to extract the maximum amount of stripe noise.
3. According to the different intensities of multi-stage stripe noise in different scales, regular terms with different sizes are selected to ensure that the stripe noise in each vertical component is removed to the maximum extent.

## 2. Algorithm Composition

Due to the specificity of stripe noises in spatial distribution, this section aims to discuss the importance of two components of the proposed method: multi-scale wavelet transform and multinomial sparse representation.

### 2.1. Multi-Scale Wavelet Transform of Infrared Images with Stripe Noises

**Figure 1:**
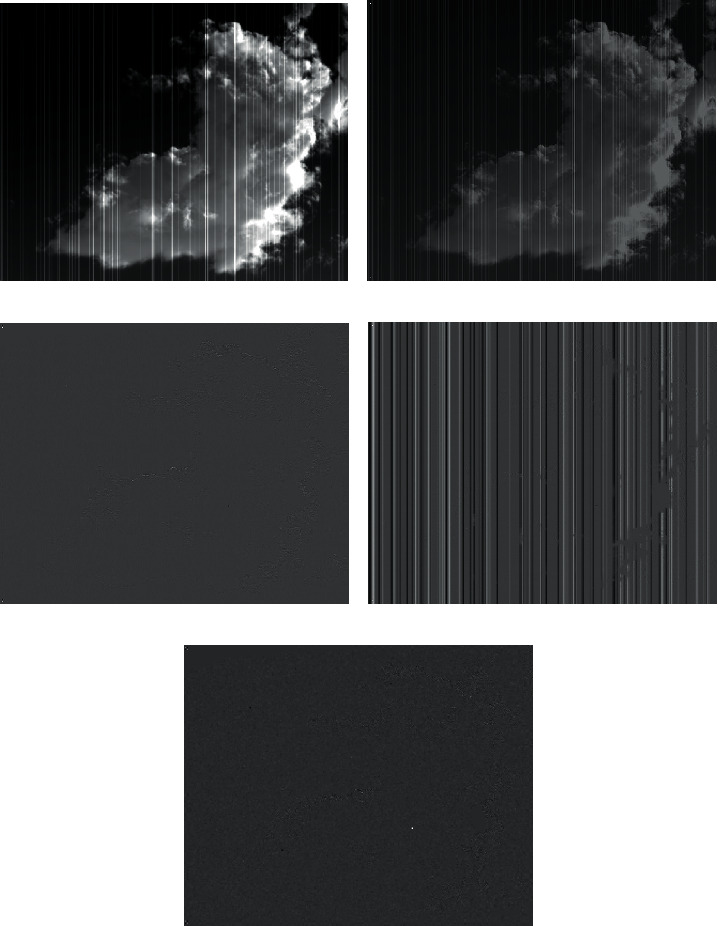
The first-level wavelet transform of the infrared image containing vertical streak noise. (a) Infrared image containing vertical stripe noise. (b) First-level approximate component. (c) First-level horizontal component. (d) First-level vertical component. (e) First-level diagonal component.

For infrared images containing vertical stripe noise, wavelet transform can extract the stripe noise and can gradually refine the image at multiple scales by local analysis of spatial frequency. The streak noise in the image can be extracted to the maximum extent.  Figure 1 shows the first-level wavelet transform of the infrared image containing vertical streak noise.

The first-level wavelet transform can be described by the following formula:

(1) $$\begin{array}{c} I.=cA_{1}.+cH_{1}.+cV_{1}.+cD_{1}, \end{array}$$

where *I*, *cA*_1_, *cH*_1_, *cV*_1_, and *cD*_1_ are the original infrared image containing vertical stripe noise, the first-level approximate component, the first-level horizontal component, the first-level vertical component, and the first-level diagonal component, respectively. “.=” denotes wavelet transform and wavelet inverse transform, not mathematically equivalent. “.+” means the set of all components in the same level, not mathematically addition.

As shown in Figure 1(d), the high-frequency stripe noises in the original infrared image are extracted into the vertical component. The remaining lower frequency stripe noises are retained in the approximate component, as shown in Figure 1(b). Then, we can extract the stripe noise from this first-level approximate component to the second-level vertical component by wavelet transform. The formula which describes second-level wavelet transform is as follows:

(2) $$\begin{array}{c} cA_{1}.=cA_{2}.+cH_{2}.+cV_{2}.+cD_{2}, \end{array}$$

where *cA*_2_, *cH*_2_, *cV*_2_, and *cD*_2_ are the second-level approximate component, the second-level horizontal component, the second-level vertical component, and the second-level diagonal component, respectively. The multi-scale wavelet transform can be described by the following formula:

(3) $$\begin{array}{c} cA_{n-1}.=cA_{n}.+cH_{n}.+cV_{n}.+cD_{n}. \end{array}$$

In practical application, the proportion of noise component of image is pretty low, and so it is difficult to observe the existence of stripe noise in the high-level vertical component after multi-scale wavelet transform. Therefore, in this paper, our proposed method is denoised only for the vertical components of all levels.

### 2.2. Multinomial Sparse Representation

**Figure 2:**
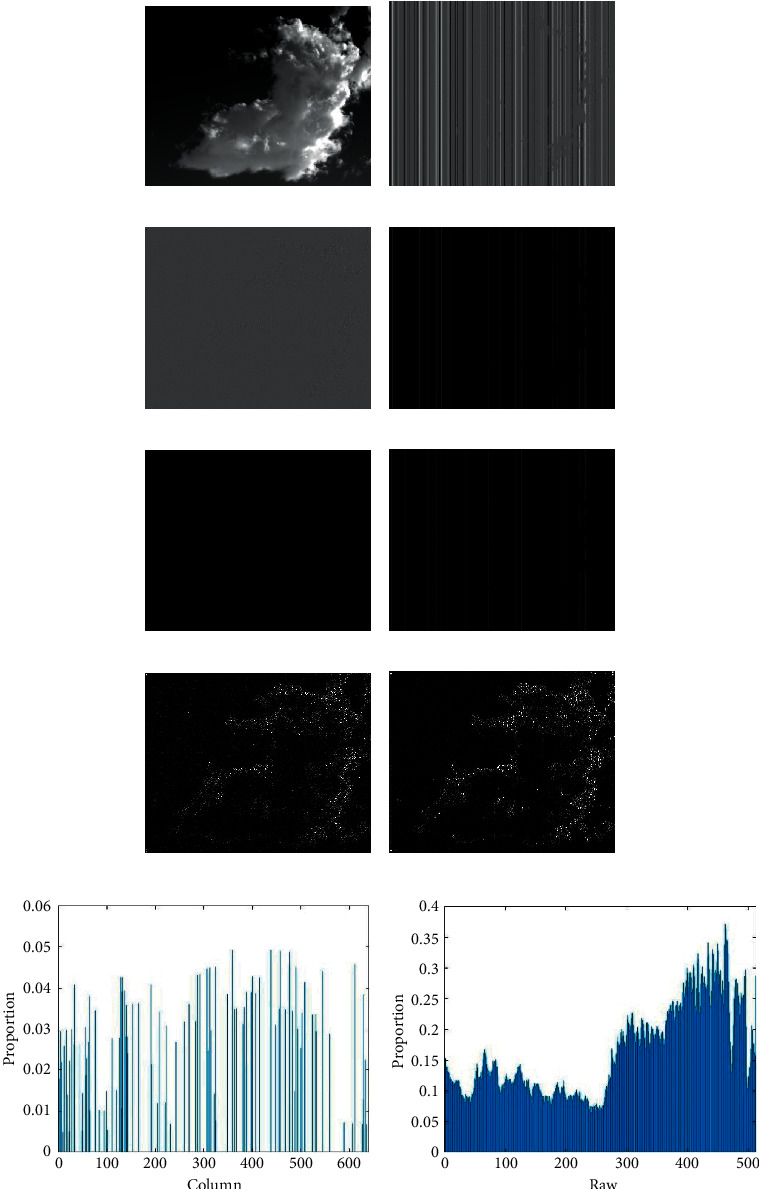
Some properties of stripe noises. (a) Ideal image. (b) The first-level vertical component of the original image. (c) Ideal first-level vertical component. (d) Stripe noises in (b). (e) Vertical gradient of (d). (f) Horizontal gradient of (d). (g) Vertical gradient of (c). (h) Horizontal gradient of (c). (i) Ratio of the L1-norm for each column of (d) in the L1-norm for the vertical gradient of (b). (j) Ratio of the L1-norm for each row of (c) in the L1-norm for the horizontal gradient of (b).

To better build a denoising model that conforms to the characteristics of streak noise, it is necessary to analyze the characteristics of streak noise and ideal vertical components from multiple angles to build the corresponding regular terms.  Figure 2 shows the difference between the ideal vertical component and the noisy vertical component by taking the first-level vertical component and the streak noise within it as an example.

#### 2.2.1. Directionality

Figure 2 shows the property differences between stripe noise in the vertical component of the original image and the ideal vertical component. From the comparison of Figures 2(e) and 2(g), we can observe that the streak noise is significantly much smoother than the ideal vertical component in terms of the gradient in the vertical direction.  Figure 2(e) also exhibits high sparsity. We constrain this sparsity of the stripe noise using the L0-norm which describes the sparsity extremely well [21, 22]. Therefore, the established regular term is as follows:

(4) $$\begin{array}{c} P_{1}\left(N_{n}\right)=d_{y}N_{n0}, \end{array}$$

where *d*_*y*_ denotes the convolution operator in the vertical direction, *N*_*n*_ denotes stripe noises in the *n*_th_-level vertical component. Models containing L0-norms with nonconvexity have difficulty in computing extrema. In contrast, the L1-norm, although slightly less descriptive of sparsity than the L0-norm, is easier to compute [23, 24]. Hence, formula (4) can be optimized as:

(5) $$\begin{array}{c} P_{1}\left(N_{n}\right)=d_{y}N_{n1}. \end{array}$$

There are many model optimization methods that use the root mean squared error or squared error as the fidelity term to protect the valid information in an image [25]. These fidelity terms are shown in formulas (6) and (7):

(6) $$\begin{array}{c} P_{2}\left(N_{n}\right)=rV_{n}-N_{n2}^{2}, \end{array}$$

Or:

(7) $$\begin{array}{c} P_{2}\left(N_{n}\right)={\left(rV_{n}^{2}-N_{n}^{2}\right)}^{1/2}, \end{array}$$

where *rV*_*n*_ is the *n*_*th*_-level vertical component of original image. *rA*_*n*_ − *N*_*n*_ is essentially the *n*_th_-level ideal vertical component *dV*_*n*_. Formulas (6) and (7) neither take into account the characteristics of ideal vertical component *dV*_*n*_ nor noise *N*_*n*_. From the comparison between Figures 2(f) and 2(h), we can conclude that the ideal vertical component is smoother in terms of the horizontal gradient. Therefore, we can adopt the smoothed horizontal gradient of the ideal vertical component as the fidelity term, which is also sparsely represented by the L1-norm:

(8) $$\begin{array}{c} P_{2}\left(N\right)=d_{x}rV_{n}-d_{x}N_{n1}. \end{array}$$

Moreover, Figure 2(j) further corroborates the strong sparsity of the ideal vertical component in the horizontal direction. Thus, formula (8) can ensure the smoothness of the denoised image in the horizontal direction.

#### 2.2.2. Structuredness

Figure 2(d) shows that the streak noise in the vertical components at all levels is with overall sparsity. We constrain this sparsity of the stripe noise using the L0-norm which describes the sparsity extremely well. Thus, we can obtain:

(9) $$\begin{array}{c} P_{3}\left(N_{n}\right)=N_{n0}. \end{array}$$

The same principle as formula (4), models containing L0-norms with nonconvexity have difficulty in computing extrema. Therefore, L1-norms are used to replace L0-norms as:

(10) $$\begin{array}{c} P_{3}\left(N_{n}\right)=N_{n1}. \end{array}$$

Moreover, Figure 2(h) further corroborates the high sparsity of the stripe noises in the ideal vertical component. Thus, formula (10) ensures that stripe noises are not excessively removed.

## 3. Methodology

In this section, the three regular terms proposed in Section 2 are generalized to propose the stripe noise removal model for infrared images. In addition, the ADMM algorithm is applied to solve this model.

**Figure 3:**
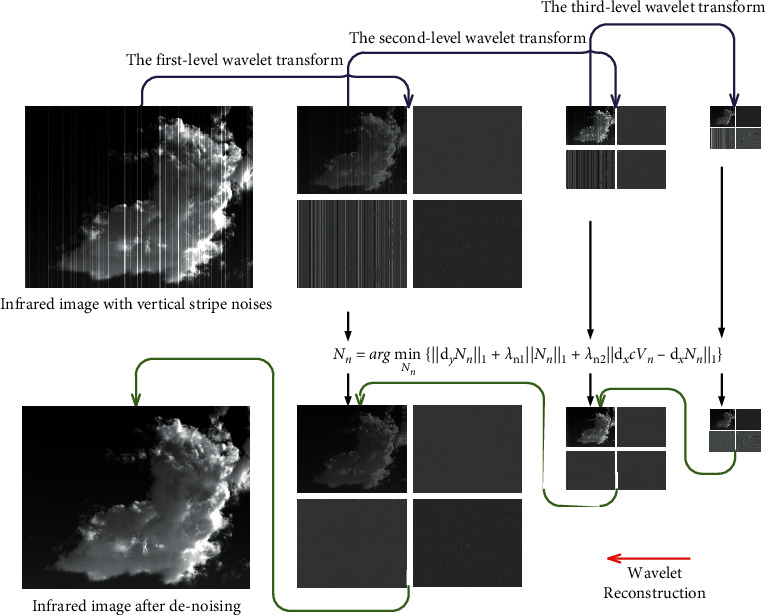
Flowchart of the proposed algorithm.

Figure 3 illustrates the flow of our proposed algorithm. Firstly, our method performs a multi-level decomposition of the infrared image using the multi-scale wavelet transform, so that the streak noise of different frequencies is extracted into different levels of vertical components. Then, these vertical components are denoised. Finally, the image is reconstructed by multiple wavelet inverse transform to obtain a clean image. The denoising of these different levels of vertical components is achieved by a multinomial sparse representation model. In this section, we will elaborate on the model and the optimization process. The process of wavelet decomposition is detailed in the reference [7].

### 3.1. Model

The previous section analyzed the characteristics of the streak noise compared to the ideal vertical component at all levels and proposed three terms *P*_1_(*N*_*n*_), *P*_2_(*N*_*n*_),  *an*  *d* *P*_3_(*N*_*n*_) for its directionality and structure. The three terms are combined to gain an optimizable model for the removal of streak noise in the *n*_th_-level vertical component:

(11) $$\begin{array}{c} N_{n}=\arg\underset{N_{n}}{\min}\left\{d_{y}N_{n1}+\lambda_{n1}N_{n1}+\lambda_{n2}d_{x}rV_{n}-d_{x}N_{n1}\right\}, \end{array}$$

where *n* denotes the level of multi-scale wavelet transform, *λ*_*n*1_ and *λ*_*n*2_ denote the parameters of the regular terms, which are assigned different weights to each term. Since the streak noise is additive noise, we extract the streak noise in the *n*_th_-level vertical component by formula (11), and then the *n*_th_-level denoised vertical component can be obtained by the following formula:

(12) $$\begin{array}{c} dV_{n}=rV_{n}-N_{n}. \end{array}$$

Finally, each level denoised vertical component *dV*_*n*_ is combined with the other unprocessed *rV*_*n*_ to perform a step-by-step wavelet inversion transform to obtain a reconstructed clean image:

(13) $$\begin{array}{c} \left\{\begin{array}{c} dA_{n-1}=.cA_{n}.+cH_{n}.+dV_{n}.+cD_{n}\\ \vdots \;\\ I_{d}=.cA_{1}.+cH_{1}.+dV_{1}.+cD_{1}\; \end{array}\right., \end{array}$$

where **I**_**d**_ denotes denoised clean image, =. denotes wavelet inversion transform.

### 3.2. ADMM Optimization

In the model optimization method, scholars often use the derivative to obtain the optimal result. However, formula (11) constructed based on the L1 parametrization is not a continuously divisible function, and it is difficult to find the extreme value. The ADMM algorithm is a more widely used method in machine learning to find the optimal solution to constrained problems, and the parts of unconstrained optimization are optimized separately. Thus, in this paper, ADMM is used to solve formula (11) and the detailed procedure is shown below.

To convert the unconstrained problem into a constrained one, we introduce three variables, namely, *G*_*n*_=*d*_*y*_*N*_*n*_,  *T*_*n*_=*N*_*n*_,  and *U*_*n*_=*d*_*x*_*rV*_*n*_ − *d*_*x*_*N*_*n*_. Then, formula (11) is converted to the formula as below:

(14) $$\begin{array}{c} \arg\underset{N_{n},G_{n},T_{n},U_{n}}{\min}\left\{G_{n1}+\lambda_{n1}T_{n1}+\lambda_{n2}U_{n1}\right\}\\ s.t.G_{n}=d_{y}N_{n},T_{n}=N_{n},\text{ and }U_{n}=d_{x}rV_{n}-d_{x}N_{n}. \end{array}$$

Following the principle of ADMM algorithm, formula (14) should be transformed into an augmented Lagrangian function:

(15) $$\begin{array}{c} \arg\underset{N_{n},G_{n},T_{n},U_{n}}{\min}G_{n1}+\lambda_{n1}T_{n1}+\lambda_{n2}U_{n1}+m_{n1}^{T}\left(d_{y}N_{n}-G_{n}\right)+m_{n2}^{T}\left(N_{n}-T_{n}\right)+m_{n3}^{T}\left(d_{x}rV_{n}-d_{x}N_{n}-U_{n}\right)+\frac{\rho_{n1}}{2}d_{y}N_{n}-G_{n2}^{2}\\ +\frac{\rho_{n2}}{2}N_{n}-T_{n2}^{2}+\frac{\rho_{n3}}{2}d_{x}rV_{n}-d_{x}N_{n}-U_{n2}^{2}, \end{array}$$

where *m*_*n*1_, *m*_*n*2_, and *m*_*n*3_ denote the Lagrange multipliers of the three constraints; *ρ*_*n*1_, *ρ*_*n*2_,  and *ρ*_*n*3_ are three penalties. Due to the concept of alternating minimization of different variables of the ADMM algorithm [26], formula (15) can be transformed into four subitems for repeated solutions as follows:

(a) *G*_*n*_ subitem
  (16) $$\begin{array}{c} G_{n}=\;\arg\underset{G_{n}}{\min}G_{n1}+m_{n1}^{T}\left(d_{y}N_{n}-G_{n}\right)+\frac{\rho_{n1}}{2}d_{y}N_{n}-G_{n2}^{2}. \end{array}$$
The formula (12) in Reference [27] describes the optimization results for the *X* in the form of the following formula:
  (17) $$\begin{array}{c} \arg\underset{X}{\min}X-B_{2}^{2}+2\lambda X_{1}. \end{array}$$
It can be directly obtained that
  (18) $$\begin{array}{c} X=\text{soft}\left(B,\lambda \right)=\text{sign}\left(B\right)\max\left(\left|B\right|-\lambda ,0\right), \end{array}$$
where sign() denotes a symbolic function whose function is to take the sign of a number. max() denotes the function to find the maximum value. Thus, formula (16) can be converted into
  (19) $$\begin{array}{c} G_{n}=\;\arg\underset{G_{n}}{\min}G_{n1}+\frac{\rho_{n1}}{2}d_{y}N_{n}-G_{n}+{\frac{m_{n1}}{\rho_{n1}}}_{2}^{2}. \end{array}$$
Based on formulas (17) and (18), the optimal solution of formula (19) is
  (20) $$\begin{array}{c} G_{n}^{k+1}=\text{soft}\left(d_{y}N_{n}^{k}+\frac{m_{n1}^{k}}{\rho_{n1}},\frac{1}{\rho_{n1}}\right), \end{array}$$
where *k* denotes the count of its iterations.
(b) *T*_*n*_ subitem
  (21) $$\begin{array}{c} T_{n}=\arg\underset{T_{n}}{\min}\lambda_{n1}T_{n1}+m_{n2}^{T}\left(N_{n}-T_{n}\right)+\frac{\rho_{n2}}{2}N_{n}-T_{n2}^{2}. \end{array}$$
Like the *G*_*n*_ problem, it works out as follows:
  (22) $$\begin{array}{c} T_{n}^{k+1}=\text{soft}\left(N_{n}^{k}+\frac{m_{n2}^{k}}{\rho_{n2}},\frac{\lambda_{n1}}{\rho_{n2}}\right). \end{array}$$
(c) *U*_*n*_ subitem
  (23) $$\begin{array}{c} U_{n}=\arg\underset{U_{n}}{\min}\lambda_{n2}U_{n1}+m_{3}^{T}\left(d_{x}rV_{n}-d_{x}N_{n}-U_{n}\right)+\frac{\rho_{3}}{2}d_{x}rV_{n}-d_{x}N_{n}-U_{n2}^{2}. \end{array}$$
It can be solved that
  (24) $$\begin{array}{c} U_{n}^{k+1}=soft\left(d_{x}rV_{n}-d_{x}N_{n}^{k}+\frac{m_{n3}^{k}}{\rho_{n3}},\frac{\lambda_{n2}}{\rho_{n3}}\right). \end{array}$$
(d) *N*_*n*_ subitem
  (25) $$\begin{array}{c} N_{n}=\arg\underset{N_{n}}{\min}m_{n1}^{T}\left(d_{y}N_{n}-G_{n}\right)+m_{n2}^{T}\left(N_{n}-T_{n}\right)+m_{n3}^{T}\left(d_{x}rV_{n}-d_{x}N_{n}-U_{n}\right)+\frac{\rho_{n1}}{2}d_{y}N_{n}-G_{n2}^{2}+\frac{\rho_{n2}}{2}N_{n}-T_{n2}^{2}+\frac{\rho_{n3}}{2}d_{x}rV_{n}-d_{x}N_{n}-U_{n2}^{2}. \end{array}$$
Formula (25) can be simplified as
  (26) $$\begin{array}{c} N_{n}=\arg\underset{N_{n}}{\min}\left\{\frac{\rho_{n1}}{2}d_{y}N_{n}-G_{n}+{\frac{m_{n1}}{\rho_{n1}}}_{2}^{2}+\frac{\rho_{n2}}{2}N_{n}-T_{n}+{\frac{m_{n2}}{\rho_{n2}}}_{2}^{2}+\frac{\rho_{n3}}{2}d_{x}rV_{n}-d_{x}N_{n}-U_{n}+{\frac{m_{n3}}{\rho_{n3}}}_{2}^{2}\right\}. \end{array}$$
Through direct derivation of formula (26):
  (27) $$\begin{array}{c} \rho_{n1}d_{Y}^{T}⊗d_{y}⊗N_{n}^{k+1}+\rho_{n2}N_{n}^{k+1}+\rho_{n3}d_{x}^{T}⊗d_{x}⊗N_{n}^{k+1}=\rho_{n1}d_{y}^{T}⊗\left(G_{n}^{k+1}-\frac{m_{n1}}{\rho_{n1}}\right)+\rho_{n2}\left(T_{n}^{k+1}-\frac{m_{n2}}{\rho_{n2}}\right)+\rho_{n3}d_{x}^{T}⊗\left(d_{x}rV_{n}-U_{n}^{k+1}+\frac{m_{n3}}{\rho_{n3}}\right), \end{array}$$
where ⊗ is the symbol that characterizes the convolution. Since it is difficult to find derivatives directly for functions containing convolution, the Fourier transform is used in this paper to transform convolution in the time domain to multiplication in the frequency domain to facilitate implementation:
  (28) $$\begin{array}{c} \left[\rho_{n1}ℱ\left(d_{y}^{T}\right).*ℱ\left(d_{y}\right)+\rho_{n2}+\rho_{n3}ℱ\left(d_{x}^{T}\right).*ℱ\left(d_{x}\right)\right].*ℱ\left(N_{n}^{k+1}\right)=\rho_{n1}ℱ\left(d_{y}^{T}\right).*ℱ\left(G_{n}^{k+1}-\frac{m_{n1}}{\rho_{n1}}\right)+\rho_{n2}ℱ\left(T_{n}^{k+1}-\frac{m_{n2}}{\rho_{n2}}\right)+\rho_{n3}ℱ\left(d_{x}^{T}\right).*ℱ\left(d_{x}rV_{n}-U_{n}^{k+1}+\frac{m_{n3}}{\rho_{n3}}\right). \end{array}$$
By left division of the matrix, we obtain
  (29) $$\begin{array}{c} ℱ\left(N_{n}^{k+1}\right)=\frac{\left[\rho_{n1}ℱ\left(d_{y}^{T}\right).*ℱ\left(G_{n}^{k+1}-m_{n1}/\rho_{n1}\right)+\rho_{2}ℱ\left(T_{n}^{k+1}-m_{n2}/\rho_{n2}\right)+\rho_{3}ℱ\left(d_{x}^{T}\right).*ℱ\left(d_{x}rV_{n}-U_{n}^{k+1}+m_{n3}/\rho_{n3}\right)\right]}{\left[\rho_{n1}ℱ\left(d_{y}^{T}\right).*ℱ\left(d_{y}\right)+\rho_{n2}+\rho_{n3}ℱ\left(d_{x}^{T}\right).*ℱ\left(d_{x}\right)\right]}. \end{array}$$
Then, the inverse Fourier transform was introduced to obtain the formula for streak noises *N*_*n*_ in the *n*_*th*_-level ideal vertical component *rV*_*n*_:
  (30) $$\begin{array}{c} N_{n}^{k+1}={ℱ}^{-1}\left\{\frac{\left[\rho_{n1}ℱ\left(d_{y}^{T}\right).*ℱ\left(G_{n}^{k+1}-m_{n1}/\rho_{n1}\right)+\rho_{2}ℱ\left(T_{n}^{k+1}-m_{n2}/\rho_{n2}\right)+\rho_{3}ℱ\left(d_{x}^{T}\right).*ℱ\left(d_{x}rV_{n}-U_{n}^{k+1}+m_{n3}/\rho_{n3}\right)\right]}{\left[\rho_{n1}ℱ\left(d_{y}^{T}\right).*ℱ\left(d_{y}\right)+\rho_{n2}+\rho_{n3}ℱ\left(d_{x}^{T}\right).*ℱ\left(d_{x}\right)\right]}\right\}, \end{array}$$
where “.*∗*” denotes the point-to-point multiplication between matrices.”./” denotes the point-to-point division between matrices. “ℱ()” stands for the Fourier transform that converts the time domain to the frequency domain; “ℱ^−1^()” represents the inverse Fourier transform that converts the frequency domain to the time domain. It is worth noting that all matrices need to be normalized before the operation. In addition, the iterative formula for Lagrange multipliers is as follows:
  (31) $$\begin{array}{c} \left\{\begin{array}{c} m_{n1}^{k+1}=m_{n1}^{k}+\rho_{n1}\left(d_{y}N_{n}^{k+1}-G_{n}^{k+1}\right)\\ m_{n2}^{k+1}=m_{n2}^{k}+\rho_{n2}\left(N_{n}^{k+1}-T^{k+1}\right)\;\\ m_{n3}^{k+1}=m_{n3}^{k}+\rho_{n3}\left(d_{x}rV_{n}-d_{x}N_{n}^{k+1}-U_{n}^{k+1}\right). \end{array}\right.. \end{array}$$
Finally, the stripe noises *N*_*n*_^*k*+1^ in the *n*_th_-level vertical component were obtained, and the denoised image *I*_*d*_ was calculated by formula (13).

## 4. Experimental Results

To demonstrate the practicality and superiority of the algorithm proposed in this paper, we designed the control experiments in this section by taking into account the three state-of-the-art methods that currently exist. These three infrared image streak noise removal algorithms are multi-scale guided filtering method (MSGF) [7], gradient equalization method based on wavelet transform (WAGE) [11], and full variational method based on guided filtering (TVGF) [14]. All image data in the experiments were actually taken. The tool used is a cooled medium-wave surface array infrared camera model TB640-CL from LUSTER. The image resolution is 640 ∗ 512.

The processing results of each algorithm need to be evaluated. There are two methods of evaluation: one is subjective evaluation, which is mainly through the human eye to observe the degree of stripe noise removal and the level of information protection in non-stripe noise regions in the image; the other is objective indexes, and three evaluation indexes that are widely recognized by researchers in this field are selected in this section, namely: NR (noise reduction), which reflects the denoising ability of the algorithm [28, 29], MRD (mean relative deviation), which characterizes the degree of information loss in the non-stripe noise region of the image after denoising [29, 30], and ID (image distortion), which reflects the fidelity level of the algorithm [15, 31]. The definitions of these three metrics are shown in formulas (32)–(34). According to their definitions, we can learn that the higher the value of NR and ID, the lower the value of MRD, and the better the algorithm is.

(32) $$\begin{array}{c} \left\{\begin{array}{c} NR=\frac{N_{0}}{N_{1}},\\ N=\sum_{i=0}^{k}\text{mean}P\left(u_{i}\right), \end{array}\right. \end{array}$$

where *N*_0_ denotes the value of *N* in the image before denoising and *N*_1_ denotes the value of *N* in the image after denoising. *u*_*i*_ represents all frequencies where the streak noise is located. *P*() denotes the power spectral density at a certain frequency.

(33) $$\begin{array}{c} MR\;D=\frac{1}{MN}\sum_{i=1}^{MN}\frac{\left|z_{i}-g_{i}\right|}{g_{i}}\times 100\%, \end{array}$$

where *i* is the number of the pixel in the image, *g*_*i*_ represents the gray value of the pixel at point *i* in the image before denoising, and *z*_*i*_ represents the gray value of the pixel at point *i* in the image after denoising. *MN* represents the resolution of the images.

(34) $$\begin{array}{c} \left\{\begin{array}{c} I\;D=S_{1}/S_{0}\\ S=\sum_{j\neq 1}^{N-1}\text{mean}P\left(u_{j}\right) \end{array}\right., \end{array}$$

where *S*_0_ denotes the value of *S* in the image before denoising and *S*_1_ denotes the value of *S* in the image after denoising. *u*_*j*_ represents all frequencies where the non-stripe noise information is located. *P*() denotes the power spectral density at a certain frequency.

The three evaluation indices mentioned above are no-reference evaluation indices, and these evaluation metrics are often used when there is no-reference image. For some experiments with reference image, it is more convincing to evaluate the algorithm using the full-reference evaluation indices, such as the peak signal-to-noise ratio (PSNR) [32] and structural similarity (SSIM) [33, 34]. PSNR can indicate the algorithm's ability to remove noise from an image, and SSIM can reflect the algorithm's ability to protect image details. Therefore, we adopt these two metrics to determine the parameters in our approach as follows.

### 4.1. Parameter Analysis

#### 4.1.1. The Optimal Number of Transform Stages

Aiming at the practical application of multi-scale wavelet transform in infrared image stripe noise removal, the following experiments with reference images are designed to explore the optimal number of transform stages in the application, so that the algorithm can achieve excellent denoising performance with the simplest possible computational procedure.

Firstly, numerous clean reference images without stripe noise are selected, and random vertical noise images with the intensity of the actual non-uniform stripe noise are generated. The stripe noise is combined with these reference images randomly and coupled into several new images containing stripe noise. Then, the multi-scale wavelet transform is applied to the reference image and the noisy image at the same time to obtain the vertical components and other components at each level. Finally, the vertical components of all levels obtained by the multi-scale wavelet transform of the reference image are replaced by the vertical components of all levels obtained by the multi-scale wavelet transform of the corresponding image with stripe noise. Then inverse transformation is carried out to achieve image reconstruction step by step.

**Figure 4:**
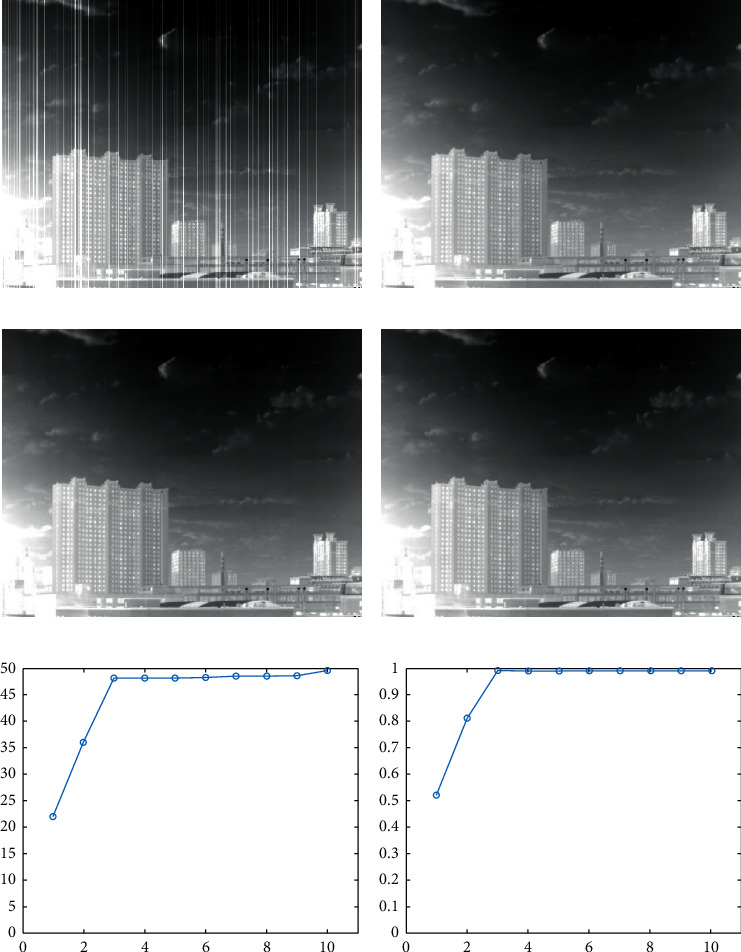
Multi-scale wavelet transform reconstruction experiments. (a) Image with streak noise. (b) Reference image. (c) Reconstructed image after three-level wavelet transform. (d) Reconstructed image after ten-level wavelet transform. (e) The relationship between the number of wavelet transform stages and PSNR. (f) The relationship between the number of wavelet transform stages and SSIM.

Figure 4 illustrates one of the multi-scale wavelet transform reconstruction experiments. Figures 4(c) and 4(d) show the reconstructed image after three-level wavelet transform and ten-level wavelet transform, respectively. We can observe that there is no significant streak noise in either image, and it is difficult to judge subjectively the quality of these two images. Moreover, in Figures 4(e) and 4(f), we are capable of concluding that after the number of wavelet transform stages exceeds three, the increase in the number of stages has little effect on the improvement of PSNR and SSIM metrics. Therefore, we let *n*=3, which allows our method to simplify the computational process while satisfying the denoising performance requirements.

#### 4.1.2. Other Parameters

**Figure 5:**
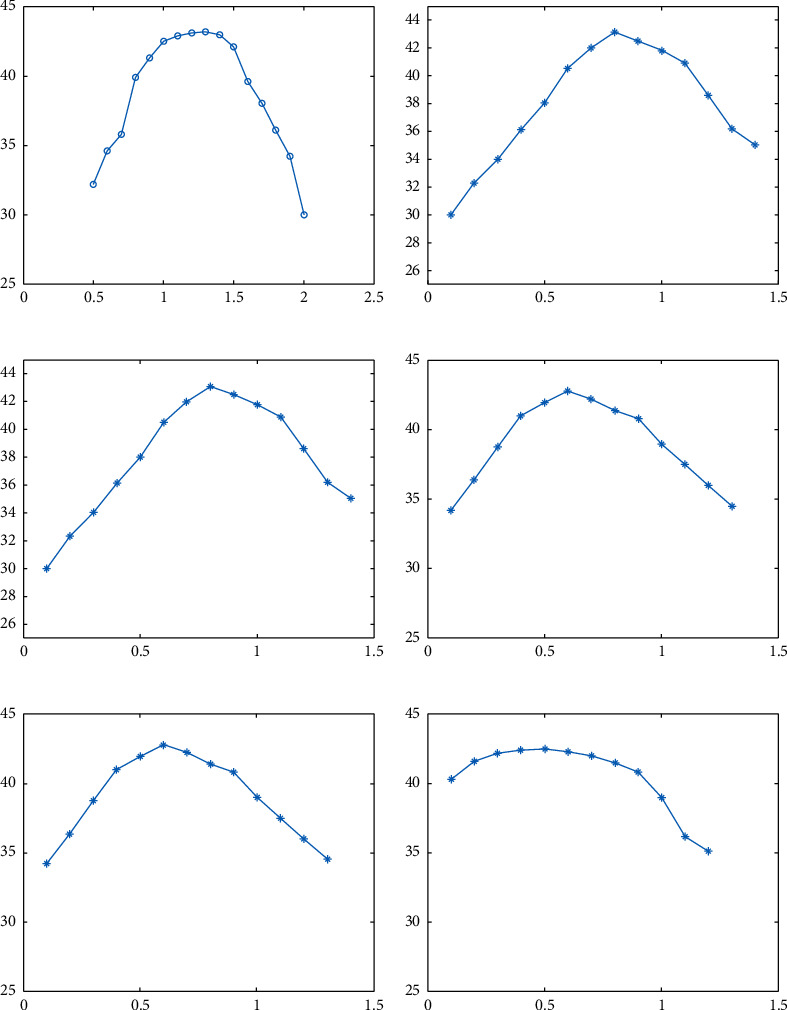
Relationship between parameters and PSNR. (a) Relationship between *λ*_11_ and PSNR. (b) Relationship between *λ*_12_ and PSNR. (c) Relationship between *λ*_21_ and PSNR. (d) Relationship between *λ*_22_ and PSNR. (e) Relationship between *λ*_31_ and PSNR. (f) Relationship between *λ*_32_ and PSNR.

According to the previous section, *n*=3. Therefore, a total of three different levels of vertical components need to be denoised. Moreover, there are six regular terms which need to be analyzed in this algorithm. Using Figure 4(a) as the experimental image and Figure 4(b) as the reference image, we explore the relationship between the coefficients of the regular terms at each level and the performance of the algorithm and seek the optimal coefficient values.  Figure 5 shows the relationship of the peak signal-to-noise ratio (PSNR) and regular terms *λ*_11_, *λ*_12_, *λ*_21_, *λ*_22_, *λ*_31_ and *λ*_32_. From the results, we can observe that there is a strong correlation between the denoising performance of the algorithm and the selection of the regular term coefficients, and there is an optimal value of the coefficients that all the algorithms need to obtain the best denoising performance. Based on the experimental results, it was set that *λ*_11_=1.3, *λ*_12_=0.8, *λ*_21_=0.9, *λ*_22_=0.6, *λ*_31_=0.7 and *λ*_32_=0.5. Similarly, we set the penalty term parameters to *ρ*_11_=*ρ*_12_=*ρ*_13_=0.20, *ρ*_21_=*ρ*_22_=*ρ*_23_=0.14, *ρ*_31_=*ρ*_32_=*ρ*_33_=0.08.

### 4.2. Experimental Contents

**Figure 6:**
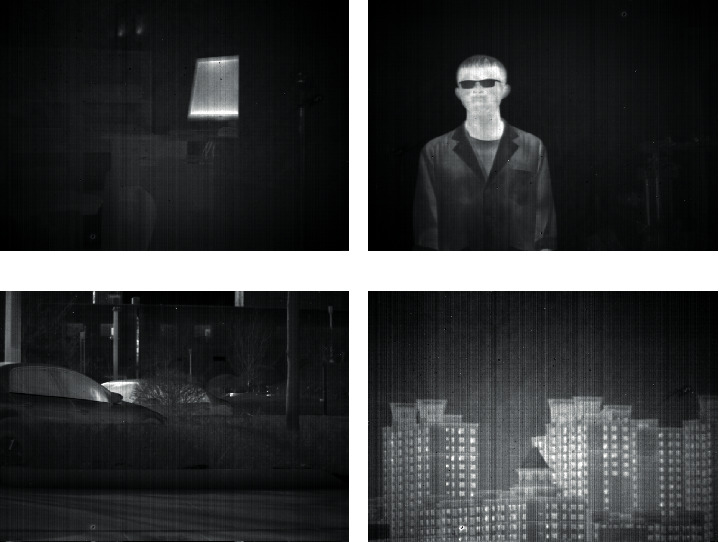
Experimental images. (a) Laboratory. (b) Person. (c) Parking space. (d) Buildings.

For the purpose of demonstrating, the practicality and superiority of the proposed algorithm, we took four infrared images with streak noise from different scenes as the experimental targets. A scene inside a laboratory is shown in Figure 6(a), which contains a computer screen with a high gray, a file cabinet, and a columnar object with a small gray difference from the background. The subject photographed in Figure 6(b) is a person, in addition to a tube with vertical characteristics that are not clearly distinguished from the background in the figure.  Figure 6(c) is taken of a parking space, consisting mainly of parked cars, trees with vertical features, and bushes with detailed information.  Figure 6(d) shows a group of distant buildings, which contains a large amount of information about horizontal and vertical directions. The brightness of the subject target and the contrast of the streak noise are different in all four images, and there are some structural features in the images. The practicality and effectiveness of the proposed algorithm can be fully demonstrated.

**Figure 7:**
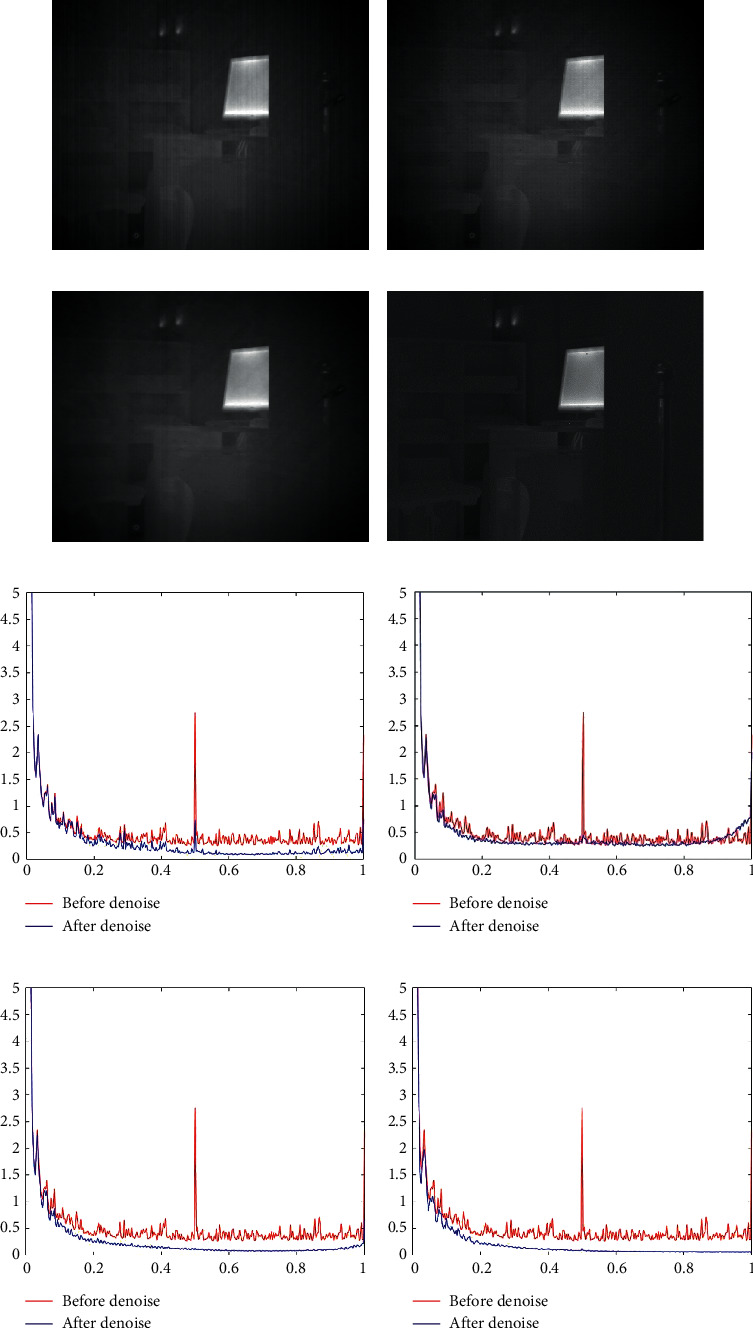
Denoising effects of different methods on an image of laboratory. (a) MSGF. (b) WAGE. (c) TVGF. (d) Our approach. (e) MSGF. (f) WAGE. (g) TVGF. (h) Our approach.

The denoising results of the four algorithms for Figure 6(a) are shown in Figure 7. As shown in Figure 7(a), the MSGF has the poorest denoising effect compared with other methods, so that some streak noises remain in the image. As shown in Figure 7(b), there are a few inconspicuous streak noises that remain in the image denoised by WAGE. As shown in Figure 7(c), the TVGF is comparable to the WAGE in terms of stripe noise removal. But in regions with low gray values, TVGF leads to significant detail loss, which indicates that it does not provide sufficient information protection in non-stripe noise regions. As shown in Figure 7(d), it can be clearly observed that the streak noise is better removed, and no over-smoothing. Figures 7(e)–7(h) show the mean power spectral density (RMPSD) of rows in Figures 7(a)–7(d) and the RMPSD of Figure 6(a), respectively, where the *x*-axis represents the normalized frequency and the *y*-axis represents the value of the RMPSD. It can be clearly observed that Figures 7(e) and 7(f) have a pulse at the frequency of a large pulse in the original image, signifying that a few streak noises have still remained. In Figures 7(g) and 7(f), it can be observed that all pulses are effectively smoothed. This phenomenon illustrates the TVGF and our approach has excellent streak noise removal capability in this infrared image.

**Figure 8:**
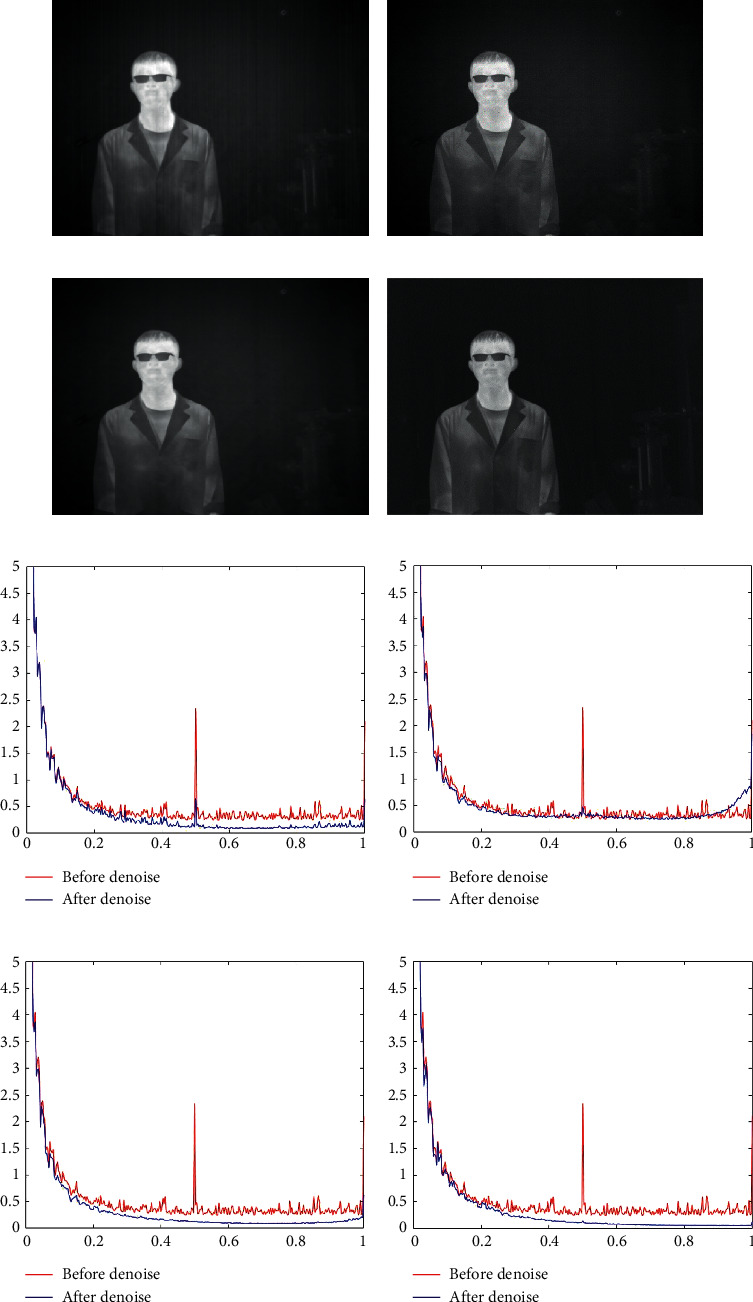
Denoising effects of different methods on an image of a person. (a) MSGF. (b) WAGE. (c) TVGF. (d) Our approach. (e) MSGF. (f) WAGE. (g) TVGF. (h) Our approach.

The denoising results of the four methods for Figure 6(b) are shown in Figure 8. In Figure 8(a), it can be clearly observed that there is a large amount of streak noise that is not removed. This can prove the weak ability of MSGF to remove the streak noise without regularity. In Figure 8(b) after WAGE denoising, it can be observed that a small amount of streak noise is still present. In Figure 8(c), it can be observed with the naked eye that there is a loss of edge information in the vertical direction of the figure. In Figure 8(d) after the denoising of our algorithm, neither significant streak noise nor information loss can be observed. In both Figures 8(e) and 8(f), we can see that spikes still exist at frequencies where streak noise often appears, which indicates that the streak noise has not been completely removed. In contrast, TVGF performs well with our method in terms of RMPSD. Combined with the above analysis, our algorithm performs the best in denoising this image.

**Figure 9:**
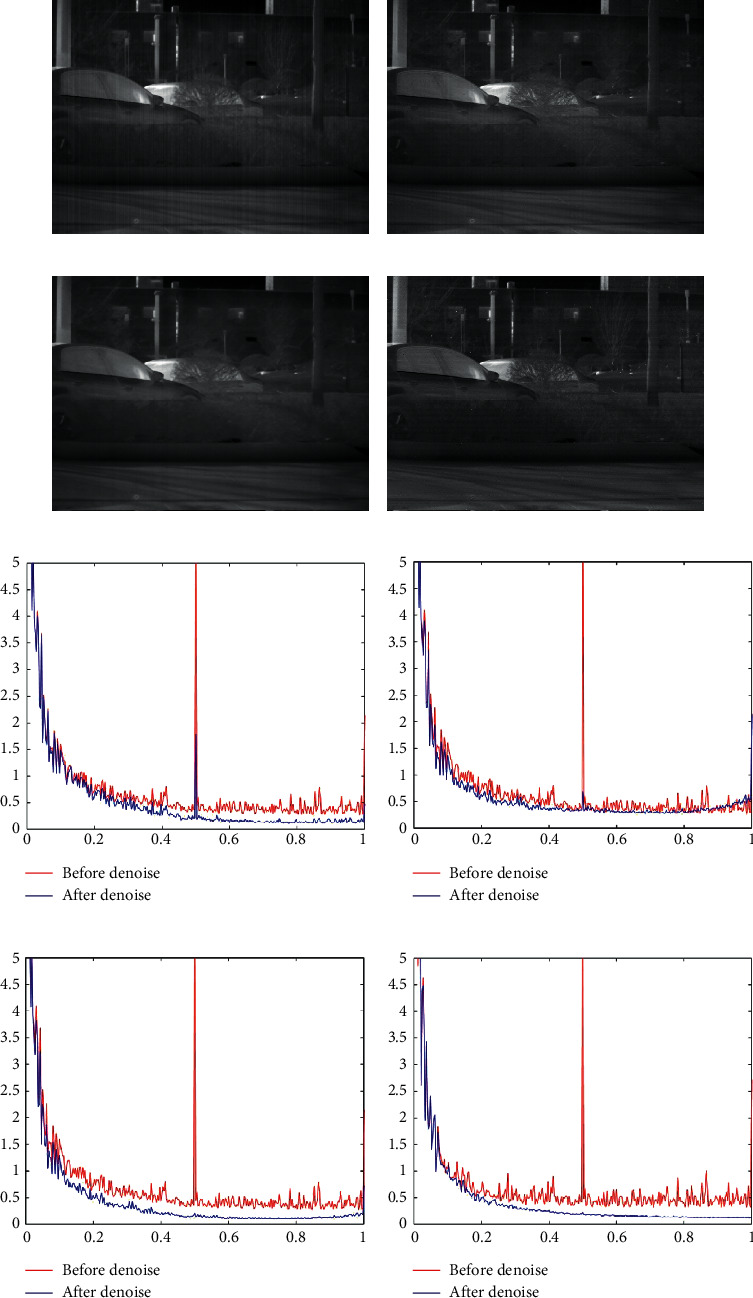
Denoising effects of different methods on an image of a parking lot. (a) MSGF. (b) WAGE. (c) TVGF. (d) Our approach. (e) MSGF. (f) WAGE. (g) TVGF. (h) Our approach.

The denoising results of the four algorithms for Figure 6(c) are shown in Figure 9. In Figure 9(a), we can clearly see that some of the noise is not removed. This shows that the MSGF is not as good as the other methods. This conclusion is further supported by Figure 9(e). In Figure 9(b), we cannot observe a clear residue of streak noises, but in Figure 9(f) we can still observe the presence of a smaller pulse, which indicates that the WAGE does not achieve the expected denoising effect. As shown in Figure 9(c), information about the details of the bush was smoothed. And Figure 9(g) implies that the stripe noises in the original image have been effectively removed. We can conclude that the TVGF has excellent streak noise removal capability, but the protection of detailed information is weak. Figures 9(d) and 9(h) illustrate the superiority of the proposed algorithm, which removes streak noises while preserving the actual information in the non-streak noise region.

**Figure 10:**
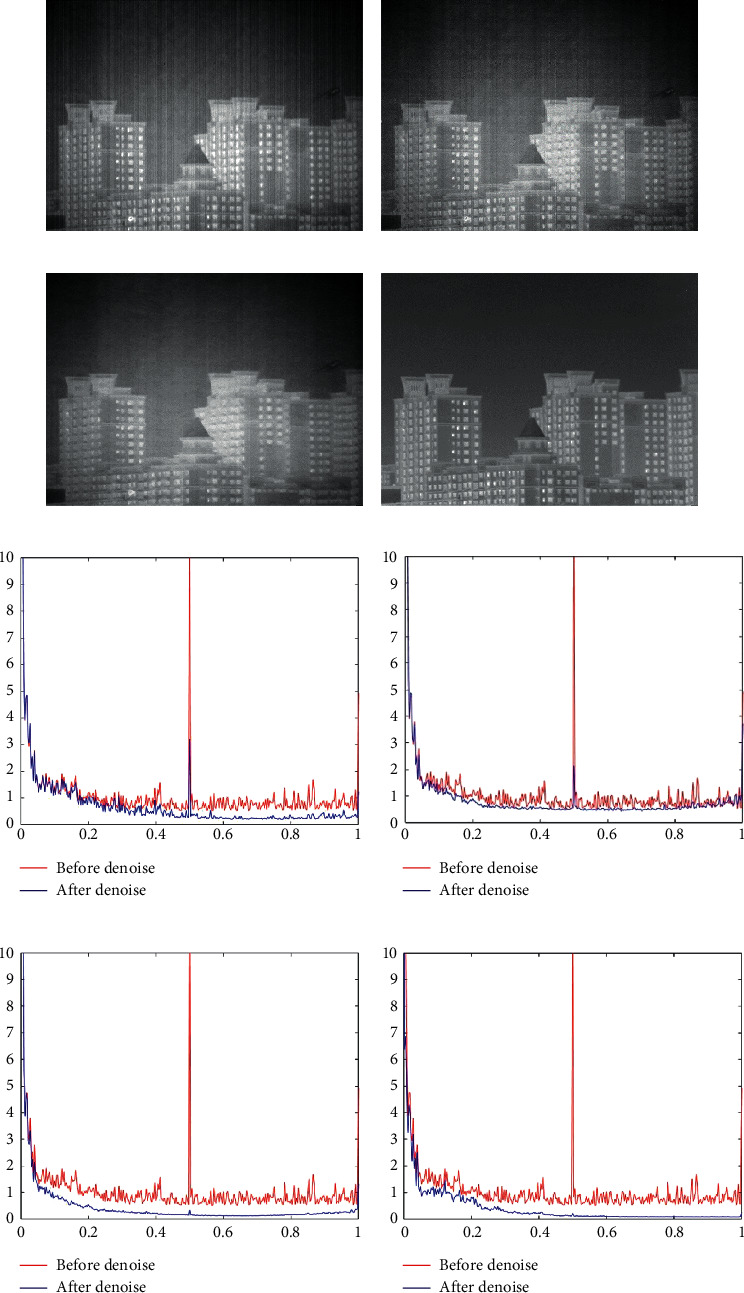
Denoising effects of different methods on an image of buildings. (a) MSGF. (b) WAGE. (c) TVGF. (d) Our approach. (e) MSGF. (f) WAGE. (g) TVGF. (h) Our approach.

The denoising results of the four algorithms for Figure 6(d) are shown in Figure 10. In Figures 10(a) and 10(b) after denoising by MSGF and WAGE, there is still significant streak noise, which directly demonstrates the shortcomings of these two methods. In Figure 10(c), we observe different degrees of blurring at the edges of all objects, which demonstrates the shortcomings of TVGF. The images processed by our method do not have the above-mentioned problems. The phenomena in Figures 10(e)–10(h) are consistent with our naked-eye observations. This shows the superiority of our method over the other three algorithms.

The NR, MRD, and ID indices of the four sets of images in Figures 7–10 are shown in Table 1. The best indices in each row are bolded.

According to Table 1, we observe that our algorithm achieves the best results in terms of NR and MRD metrics for all images, indicating that our algorithm has excellent streak noise removal capability with the preservation of non-streak noise information. As for the ID, although our algorithm did not obtain the best results, combined with the subjective analysis in Figures 7–10, our algorithm did not cause significant distortion and the ID value tends to be close to 1. These results testify that our approach is excellent in the removal of stripe noises.

## 5. Conclusion

This paper proposes a stripe noise removal algorithm for infrared images, combining multi-scale wavelet transform and multinomial sparse representation. The proposed algorithm constructs the denoising model following the differences in directionality and structure between the stripe noise and the vertical components at each level and chooses the L1-norm which describes the sparsity well. Aimed at protecting pixels in the non-striped noise region, the MSWT can reasonably separate information in the vertical direction from information in other directions, which provides maximum protection for non-striped information in the infrared image. The ADMM algorithm for solving the model is described in detail. Finally, a large number of experiments are designed to illustrate the practicality and effectiveness of the proposed algorithm. However, there are still many difficulties in the streak noise removal domain, such as stripe noises in the oblique direction. In the future, we will focus on the investigation of the oblique direction stripe noises in infrared images and optimize our method to remove them.

## Figures and Tables

**Table 1:** Indices of different methods on different images.

Image	Indices	MSGF	WAGE	TVGF	Our approach
Laboratory	NR	2.23	2.60	3.57	**3.72**
	MRD (%)	4.01	3.23	4.23	**3.15**
	ID	**0.999**	0.991	0.980	0.993

Person	NR	2.02	2.51	3.58	**4.05**
	MRD (%)	3.72	2.95	4.53	**2.92**
	ID	**0.999**	0.992	0.975	0.995

Parking lot	NR	2.27	2.78	3.56	**3.76**
	MRD (%)	3.07	2.86	3.59	**2.67**
	ID	**0.999**	0.993	0.982	0.994

Buildings	NR	2.29	2.79	3.45	**3.72**
	MRD (%)	4.36	3.94	5.47	**3.69**
	ID	**0.999**	0.991	0.971	0.994

## Data Availability

The data used to support the findings of this study are available from the corresponding author upon request.
